# Study protocol for the Rainbow Austrian Longitudinal Family (RALF) study: a longitudinal, multi-method, multi-rater investigation of risk and resilience factors in Austrian LGBTQ+ parent families

**DOI:** 10.1186/s40359-025-02828-4

**Published:** 2025-05-26

**Authors:** Betty Geidel, Magdalena Siegel, David Steyrl, Abbie E. Goldberg, Guy Bodenmann, Martina Zemp

**Affiliations:** 1 Department of Clinical and Health Psychology, University of Vienna, Waechtergasse 1/504, Vienna, 1010 Austria; 2 Department of Cognition, Emotion, and Methods in Psychology, University of Vienna, Vienna, Austria; 3 Department of Psychology, Clark University, Worcester, MA USA; 4 Department of Psychology, University of Zurich, Zurich, Switzerland

**Keywords:** Lesbian, gay, bisexual, transgender, queer parent families, Gender identity, Sexual orientation, Children and adolescents, Community-based participatory research, Intersectionality, Observational data, Minority stress, Machine learning, Clinical psychology

## Abstract

**Background:**

Research on LGBTQ+ parent families is evolving to include a growing range of family systems, identities, methodologies, and topics. However, studies that examine minority-specific risk and resilience factors and their associations with within-family processes remain scarce, particularly outside a US-American context. Addressing these research gaps quantitatively poses challenges for researchers, because traditional modelling techniques based on (generalized) linear models are not ideally suited to capture the complexity and intersectionality of family experiences. Within this study protocol, we introduce the Rainbow Austrian Longitudinal Family (RALF) study. Its main goal is to comprehensively investigate general and minority-specific factors that affect the well-being of LGBTQ+ parent family members in Austria.

**Methods:**

RALF is a three-wave, longitudinal study over two years that examines risk and resilience factors at the individual, couple, and family level using a multi-method, multi-rater approach. We will assess child adjustment outcomes across three child cohorts, parental mental health, and family processes across three annual data waves through online questionnaires. We aim to recruit *N* = 150 LGBTQ+ parent families from a variety of family forms and with various identities residing in Austria. A focal sample (target *n* = 60) will additionally participate in observational, video-based assessments. Our participatory research approach aims to actively engage community members and stakeholders throughout the study. A community advisory board ensures that the study reflects the lived experiences of LGBTQ+ parent families adequately, while community events and accessible dissemination strategies for study results, such as the open access data explorer *ExploRALF*, support community engagement and facilitate the dissemination and discussion of results. Data will be analyzed using machine learning-based approaches designed to capture complex, non-linear interactions, which are ideally suited to model intersectional experiences of LGBTQ+ parent families.

**Discussion:**

The RALF study is the first prospective study to comprehensively investigate minority-specific risk and resilience factors in Austrian LGBTQ+ parent families. Findings have the potential to fill key research gaps, inform policy, and guide clinical practices that support LGBTQ+ parent families.

Research on lesbian, gay, bisexual, transgender (trans), and queer (LGBTQ+) parent families has made substantial progress in understanding both the strengths these families possess as well as challenges they face. Over the last decades, this line of research has evolved significantly [1–4]. In its early stages, scholars needed to provide evidence of LGBTQ+ parent families’ ability to “perform” comparably to cisgender heterosexual families across key domains (e.g., child adjustment, intellectual development, parenting, and family functioning) – a step necessary to counter the biases present in society and to affirm the strengths of these families based on empirical evidence [1–7]. This phenomenon, termed “deficit-comparison perspective” [7], positioned the cisgender heterosexual nuclear family as the gold standard against all other family structures [1, 8], while focusing research primarily on lesbian and gay (LG) parent families [6, 9].

While this approach played an essential role in challenging prejudice in the socio-political climate of the time, it came with profound consequences for the development of the field: In seeking to counteract societal prejudice, in which dissimilarities were frequently met with skepticism, researchers operated within the constraint of needing to demonstrate sameness [3]. This hindered the exploration of distinctive strength and risk factors of as well as diversity within LGBTQ+ parent families [1, 3], and thus contributed to the conceptualization of lesbian (and gay) parent families as a homogeneous group when contrasted with cisgender heterosexual families [3, 10].

Another issue of structural barriers to this research field pertains to scholars working in it, particularly those identifying as LGBTQ+, who have long faced and continue to face significant professional and personal challenges [11]. These include the legitimacy of their work being called into question and their contributions being devalued [5, 11]. Many encounter barriers to publication opportunities in high-impact journals [5, 11, 12], along with limited prospects for funding, frequently influenced by political and social climates [5, 12]. As a result, career prospects remain precarious [5]. Given these persistent obstacles, the contributions of LGBTQ+ researchers are all the more remarkable. Their work has not only expanded academic knowledge but has also played a crucial role in fostering and societal acceptance of LGBTQ+ individuals and families. The scientific contributions provided by these studies have been instrumental in shaping the social, legal, and political discourse on LGBTQ+ parent families, and helped advance policy changes that led to the recognition and legal protection of LGBTQ+ parent families, predominantly in English-speaking and Western-European countries [13–17]. Building on these foundations, contemporary LGBTQ+ parent family research now has the opportunity to grow beyond these roots [15, 18]. Less constrained by a comparative perspective, scholars are increasingly exploring a broader spectrum of questions, and starting to discover the unique strengths, challenges, and dynamics that reflect the experiences of LGBTQ+ parent families [5].

This study protocol outlines the Rainbow Austrian Longitudinal Family (RALF) study, which will investigate LGBTQ+ parent families’ well-being within the context of shifting societal attitudes, legal advancements, and emerging research, considering both individual outcomes and relational dynamics within the family unit. Our research aims to address questions that reflect the lived experiences of Austrian LGBTQ+ parent families and contribute to theoretical advancements in the field by expanding on minority stress and resilience research, as outlined below. Employing an intersectional and community-based participatory research (CBPR) approach, the RALF study aims to build on and honor the foundational research that shaped the field, while seeking to advance our theoretical understanding of LGBTQ+ parent families and their lived experiences in an Austrian context.

## LGBTQ+ parent families: parenting with pride and peril

The growing scope of research within LGBTQ+ parent family research is reflected in the diversification of studied populations and topics, alongside advancement of both theory and methodology. One key area of growth for the field remains the development, evaluation, and integration of theoretical frameworks specifically tailored to LGBTQ+ parent family research [1, 7, 12, 15, 19, 20]. In its earlier stages, the field had little chance to establish such frameworks due to the necessity of providing answers to questions posed by public discourse. Thus, theoretical bases were often grounded in cis-heteronormative deficit models [1]. Over time, researchers began extending existing theories, for instance using family processes (e.g., parental relationships) or external influences (e.g., stigma), while frameworks that specifically take LGBTQ+ parent families into focus have only begun to emerge [1].

One prominent framework, the *minority stress theory* (MST) has been widely used to explain the persisting mental health disparities faced by LGBTQ+ individuals (e.g., [21–24]). In essence, this theory posits that individuals belonging to a sexual or gender minority experience specific stressors and stress processes (i.e., minority stressors and minority stress) related to their minority status that may have a cumulative effect on their mental, and to a lesser extent, physical health [25, 26]. Through elevations in general risk factors (e.g., social isolation, rumination) [27], these minority-specific stressors are assumed to transdiagnostically account for the higher prevalence rates of mental disorders (e.g., depression, anxiety, stress-related disorders, suicidality) found in LGBTQ+ populations [24]. According to MST, these stressors fall within a distal-to-proximal continuum. Distal stressors refer to external factors, such as discrimination and violence. Through cognitive appraisal, they may develop into proximal stressors and stress processes, such as internalized stigma (i.e., shame and negative feelings related to one’s sexual orientation or gender identity), identity concealment (i.e., hiding one’s identity from others), and expectations of and sensitivity to rejection [28]. The influence these stress processes have on mental health outcomes is shaped by coping mechanisms and social support [28].

Recently, efforts have been made to expand the model to gender minorities [29] and integrate relational systems (e.g., [7, 30–32]), such as intimate relationships (e.g., relationship concealment) [33] and the family (e.g., [31]), into this theory. One such example are recent adaptations to MST by Hendricks and Testa [29] that specifically highlight adverse experiences around gender identity and expression, such as gender non-affirmation, as specific stressors. This adaptation aims to address the unique experiences of trans and non-binary individuals, while acknowledging that the experiences of these intersecting groups are, in many ways, shared [29, 34].

Another notable example of an extension of the MST [28, 35, 36] is the inclusion of couple-level minority stress, which formalizes minority-specific stressors and stress processes equivalents at the couple level, such as stress contagion between partners [33]. Evidence supports that couple-level stressors are distinct from individual-level stressors and play a critical role in shaping the health and well-being of sexual minority individuals across both levels [33, 37–39]. By integrating role-based (couple-level) and identity-based (individual) stressors [37], this framework offers a comprehensive understanding of minority stress and highlights the interconnectedness of stress across these levels. Addressing the interaction between levels is essential to fully understand and capture the unique stress faced by sexual (and gender) minority populations [37].

Building on these expansions, scholars emphasize the need to further develop existing frameworks to examine relational contexts at multiple levels, beyond individual or couple-level minority stress [19, 30, 33, 37]. A family-level approach could provide valuable insights on how minority-specific risk and resilience factors operate within LGBTQ+ parent families. Parental experiences of minority stress may not only affect their own well-being or the couple level but also shape family dynamics in ways that influence all members of the household. For instance, child adjustment may be affected directly (e.g., by bullying due to being a member of an LGBTQ+ parent family) and indirectly (e.g., minority stress affecting parental mental health, which in turn affects family functioning and child adjustment). This analogously follows the tenets of the well-established family stress model [40], which assumes similar mediational mechanisms for financial stress and poverty and has been extensively tested using cross-sectional and longitudinal designs [40].

In response to this need for theoretical expansion to the family level, Siegel and Zemp [31] introduced a family-level minority stress model, that this study draws upon to investigate its application and further explore minority stress at the family level. The model highlights three key points: (i) minority stress can be mapped to the family level, (ii) minority stress is associated with family outcomes, and (iii) children may experience family-level minority stress [31]. By applying this model to RALF, we intend to gain empirical insights into family-level minority stress in LGBTQ+ parent families and its impact on family processes.

While minority stress centers the challenges faced by LGBTQ+ individuals, couples, and families, the concept of minority-specific resilience factors focuses on their navigation of hardship and maintenance of well-being [7]. Research demonstrates that LGBTQ+ parent families display a strong capacity for resilience in the face of adversity [6]. Building on this, Prendergast and MacPhee [7] advocate for further investigation into the unique factors that positively influence the well-being of LGBTQ+ parent families, including parenting and child outcomes. Their model of LG family resilience implicitly highlights the importance of examining resilience factors on relational levels – individual, couple, and family – as interconnected aspects [7]. Grounded in minority stress theory [28, 35, 36], the model conceptualizes the ways in which families navigate challenges, and the factors that act to stabilize the family and mitigate adverse outcomes [7]. Importantly, it underscores that resilience is not static, but shaped by a dynamic interplay of variables including risk and protective factors that support family well-being [7].

Despite this conceptual advancement, there remains a clear need for systematic empirical research on family-level resilience factors [19] and a broader understanding of the strengths of LGBTQ+ parent families [7]. Positive, minority-specific identity factors have been identified as possible contributors to resilience and well-being in sexual minorities [41] and have been linked to relational outcomes [42]. While previous studies have explored individual and couple-level positive identity factors regardless of parenting status [41–47], how these factors interact within the broader family system, such as their impact on child adjustment, remains largely unexplored [7]. This is a crucial limitation, as children in these families have been found to thrive even within stigmatizing environments [2, 48], and resilience within LGBTQ+ individuals in general has been conceptualized as emerging from experiences with marginalization or stigma [28].

Moving beyond minority-specific factors, within-family processes, such as supportive family relationships, positive parenting, or dyadic coping (i.e., joint coping efforts and mutual support in couples coping with stress) [49, 50] have long been considered influential factors to family well-being in the field of general family psychology [51–56]. For example, the quality of the interparental relationship affects their ability to coparent [55, 57]. Coparenting refers to the parents’ cooperation, coordination, and mutual support in childrearing [58]. Furthermore, supportive parenting and a strong parenting alliance are key factors in promoting child well-being generally [59] and in LGBTQ+ parent families, with higher levels of support linked to better child adjustment and fewer internalizing and externalizing problems [60–62]. Conversely, negative parenting dynamics, such as competition between parents, have been identified as significant predictors of child behavior problems [61].

Based on this, we assume that within-family processes represent key mechanisms mediating the links between risk and resilience factors in LGBTQ+ parent families and family as well as child outcomes. While self-reports provide valuable insights into these processes, observational methods offer a unique opportunity to capture the subtle nuances of family dynamics. Observational data are generally considered a gold standard for investigating family processes, because observed interactions and behaviors accurately reflect processes frequently repeated in everyday family life (e.g., [60, 63]). Despite their potential, however, there are, to our knowledge, only a few observational studies examining family dynamics in LGBTQ+ parent families [60, 61].

In addition to processes *within* the family system, previous research has shown that external, contextual influences are also highly relevant in understanding the well-being of LGBTQ+ parent family members [7, 48, 64]. These include, for instance, the families’ neighborhood, workplaces, and school environments [7, 65–67]. Moreover, societal attitudes, particularly negative attitudes towards LGBTQ+ parenting, can impair coparenting, as internalized stigma may inhibit cohesion and a positive connection between parents [68]. Based on these findings, we argue that research in this field must consider both general and minority-specific intra- and extra-familial risk and resilience factors, along with family processes within LGBTQ+ parent families as potential key mechanisms that may explain how those factors affect the well-being of all family members.

## Navigating nuance through advanced statistical methods: intersectionality within quantitative LGBTQ+ parent family research

Research has recently begun to explore the concept of intersectionality within the context of LGBTQ+ parent families [15, 18, 31, 69–71], recognizing that this perspective is essential for capturing the complexities that shape these families’ experiences. Given its significance, scholars continue to call for even greater attention to intersectional approaches in future research [18, 71, 72]. Originating from Black feminist scholarship, thus being informed by the experiences of Black women at the intersection of racism and sexism [73], intersectionality frameworks seek to understand and appreciate how intersecting identity dimensions shape individual experiences through similarly intersecting systems of power on the structural level [74]. It highlights how multiple social categories (e.g., gender identity, sex assigned at birth, sexual orientation, race, ethnicity, socioeconomic status) interrelate on an individual level [73, 75]. These categories are assigned marginalization or privilege through multiple interlocking (power) systems (e.g., heterosexism, cissexism, racism, classism) on a structural level [76], driving intersectional inequalities on an individual level [74, 76].

Intersectional experiences form at the basis of a complex structure of privilege and oppression, with identity dimensions influencing each other in intricate ways. While qualitative and mixed-method approaches are particularly suited for capturing intersectional experiences, traditional quantitative methods, mostly reliant on linear models, struggle to account for the complex, often non-linear interactions that are indispensable to examine intersectionality [75, 77]. Statistically, intersectionality translates into potentially high-dimensional and possibly non-linear associations between variables and thus represents a formidable challenge for the selection of suitable methodological approaches within quantitative designs [15, 75–77]. This complexity increases even further when considered in the context of family research and systems [30, 31, 78, 79]. This can be attributed, for instance, to intersecting systems of structural oppression at multiple levels within the family and the presence of interdependent family members as raters [19, 30, 78, 79]. To address these challenges, researchers are increasingly advocating for innovative modeling techniques that capture these non-linear associations between variables as well as high-dimensional interactions [80, 81]. Non-linear machine learning (ML) methods show great promise for modeling intersectionality in quantitative research by effectively handling these complexities [80–83]. In particular, gradient boosted decision trees (GBDT), a well-established ML method, are used for their efficiency, accuracy, robustness against multicollinearity, and their ability to model complex, non-linear and high-dimensional associations between variables [84]. There has been an increase in the utilization of ML techniques in recent psychological research [85]. However, to date, the application of these techniques within the field of general family research [86] and LGBTQ+ research [87] remains limited. In order to address this gap and effectively model intersectional experiences in LGBTQ+ parent families, we will implement ML methods within the RALF study.

## Building bridges with community-based participatory research: reaching underrepresented identities and family forms

Historically, a significant portion of LGBTQ+ parent family research has centered on monosexual identities [1, 3, 4, 6, 9, 18], often classifying individuals only based on the gender of a partner [88]. While early works on LGBTQ+ parent families have primarily focused on those with lesbian and gay parents, the focus has recently shifted to examining less studied and harder-to-reach populations (e.g., asexual, trans, bisexual and other plurisexual identified parents) [18, 72, 89–92]. This expansion of research also entails the investigation of various pathways to parenthood – beyond parenthood in the context of a previous heterosexual relationship [72, 89–92] – including, but not limited to step-parenting, adoption, surrogacy, and reproductive technologies [72, 93, 94]. Despite these strides, there remains considerably more to discover to advance the field with a deeper understanding of the unique challenges, strengths, and perspectives of LGBTQ+ parent families across a range of identities and backgrounds. To this end, scholars have called for a transition from research *on* LGBTQ+ parent families to research *with* this community (e.g., [95]).

Community-based participatory research (CBPR) represents a promising approach in this respect. CBPR aims to involve community engagement in every stage of the research process, including the development of the study design, participant recruitment, and interpretation and dissemination of findings [96]. Hence, by integrating diverse experiential backgrounds in research, CBPR offers a valuable opportunity to enhance the relevance and the applicability of research findings to the relevant communities [96–101]. Building an active, democratic, and collaborative research relationship to achieve a mutual exchange of knowledge is a key benefit of CBPR [96, 101]. Its application is particularly promising in research about health disparities with marginalized communities [99, 102, 103], such as LGBTQ+ individuals and their families, who repeatedly face inequity and adversity in various aspects of their daily lives [2]. CBPR counters these lived experiences with the basic principles of inclusivity, cooperation, mutual respect, and trust [98, 100, 102, 103]. To further explore this approach within the context of LGBTQ+ parent family research, we will adopt a CBPR approach, actively involving these families within the RALF study.

Community Advisory Boards (CABs), as implemented within the RALF study, represent a specific component of CBPR. Typically, such boards consist of community members (e.g., LGBTQ+ parents) and other stakeholders (e.g., human rights advocates) who provide feedback throughout the research cycle to represent the interests of the community and help maintain ethical and scientific integrity [99, 101, 104, 105]. Beyond facilitating research, CABs harbor the potential to mitigate power asymmetries [98, 106]. It is, therefore, a highly valuable, yet challenging, undertaking that requires a high degree of sensitivity and care from all those involved.

## LGBTQ+ parent families in Austria: an underresearched population

The current body of knowledge concerning risk and resilience factors in LGBTQ+ parent families is primarily based on research from the United States and some Western-European countries [64]. Literature on the subject in Austria is, by contrast, limited [107–109]. This is a significant knowledge gap, given that studies, albeit non-representative, suggest that a substantial number of LGBTQ+ individuals in Austria are raising children [14, 108].

Recent legal and social developments in Austria, including the introduction of legal adoption of stepchildren by same-gender couples (second parent adoption; 2013), joint adoption (2016), and marriage equality (2019) [110, 111], have likely even resulted in a rise in the number of children growing up in LGBTQ+ parent families. However, representative data remain limited [108]. At the same time, there has been an overall increase in social acceptance of LGBTQ+ individuals and their families in Austria [112]. While historical and sociolegal contexts vary across countries, thus precluding comparisons [107], international data support the idea that the aforementioned legal and societal changes could further encourage the formation of LGBTQ+ parent families – including in Austria [113, 114].

Against this backdrop, more research is needed into the specific experiences, resources, and mental health of all members of LGBTQ+ parent families in Austria. We know particularly little about the well-being and development of children of different ages growing up in Austrian LGBTQ+ parent families over a longer period. A recent systematic review revealed that there is no published longitudinal psychological family research regarding LGBTQ+ parent families specific to the Austrian context [107].

## The RALF study

Drawing upon extant research in this field, the RALF study seeks to provide a three-wave longitudinal assessment of family process variables and a broad array of individual and family outcomes in LGBTQ+ parent families, including well-being, mental health, relationship satisfaction, parental stress, coparenting, and child adjustment. In order to achieve a comprehensive understanding of families’ experiences, we investigate sociodemographic characteristics (e.g., sexual orientation, gender identity, family structure), minority-specific risk and resilience factors (e.g., family minority pride and stress), within-family processes (e.g., dyadic coping, coparenting), and extra-family dynamics (e.g., school and neighborhood climate) across the three annual data waves.

Our multi-rater, multi-method approach combines observational, and questionnaire data collected from a diverse set of families within three age cohorts: families with children in early childhood (2;0–5;11 years), middle childhood (6;0–10;11 years), and adolescence (11;0–17;11 years). Using non-linear, machine learning-based techniques, we will model complex intersectional experiences and variable interactions to accurately capture the lived experiences of LGBTQ+ parent families. Furthermore, there is a critical need for comprehensive data on LGBTQ+ parent families in Austria that calls for a CBPR approach to provide insights into the community’s (country-) specific needs in the absence of empirical data to date. Our CBPR components will include collaborative work with a Community Advisory Board (i.e., the RALF CAB) to guide and inform data collection, a data explorer (i.e., the *ExploRALF*) to disseminate our findings in an accessible and low-threshold way, and public events to strengthen community connection.

## Research questions

The project will address a broad set of main research questions (RQs) and hypotheses (H), of which we only present selected examples in this protocol. Individual RQs for future publications, their respective hypotheses and analytic procedures will be preregistered separately.

RQ 1: Are risk and resilience factors as well as moderators at the parent, child, couple, and family level associated with child adjustment?

H 1: Risk and resilience factors as well as moderators at the parent, child, couple, and family level are cross-sectionally associated with child adjustment at each data wave (W1, W2, W3).

RQ 2: Are parental minority-specific risk and resilience factors prospectively associated with parental mental health and child adjustment?

H 2: Parental minority-specific risk and resilience factors at W1 are associated with parental mental health at W2, which, in turn, predicts child adjustment at W3.

RQ 3: Are parental minority-specific risk and resilience factors prospectively associated with dyadic and triadic family processes and child adjustment?

H 3: Parental minority-specific risk and resilience factors are associated with family processes assessed at the consecutive wave and these family processes are associated with child adjustment assessed at the consecutive wave.

## Methods

### Study design and study framework

#### Study design

Our study design comprises three waves of national longitudinal multi-method, multi-rater assessments, including self-report questionnaires and observational data spanning a duration of 3.5 years in total (data collection over two years). Questionnaire data will be obtained using online surveys administered within different modules for different subpopulations (e.g., parents in a relationship) and divided into three child age cohorts (see section Sample and eligibility criteria). These will be completed at each of the three time points of the data waves (W1, W2, W3, each with a 1-year interval in between time points) by parents and adolescents (oldest age cohort: 11;0–17;11 years). Observational data will be collected exclusively during the first and third data wave (W1, W3) with a focal subsample. This subsample will consist of our youngest age cohort, i.e., families with at least one child aged 2;0 and 5;11 years (cohort *early childhood*).

#### Study framework: assessing risk and resilience in LGBTQ+ parent families

**Fig. 1:**
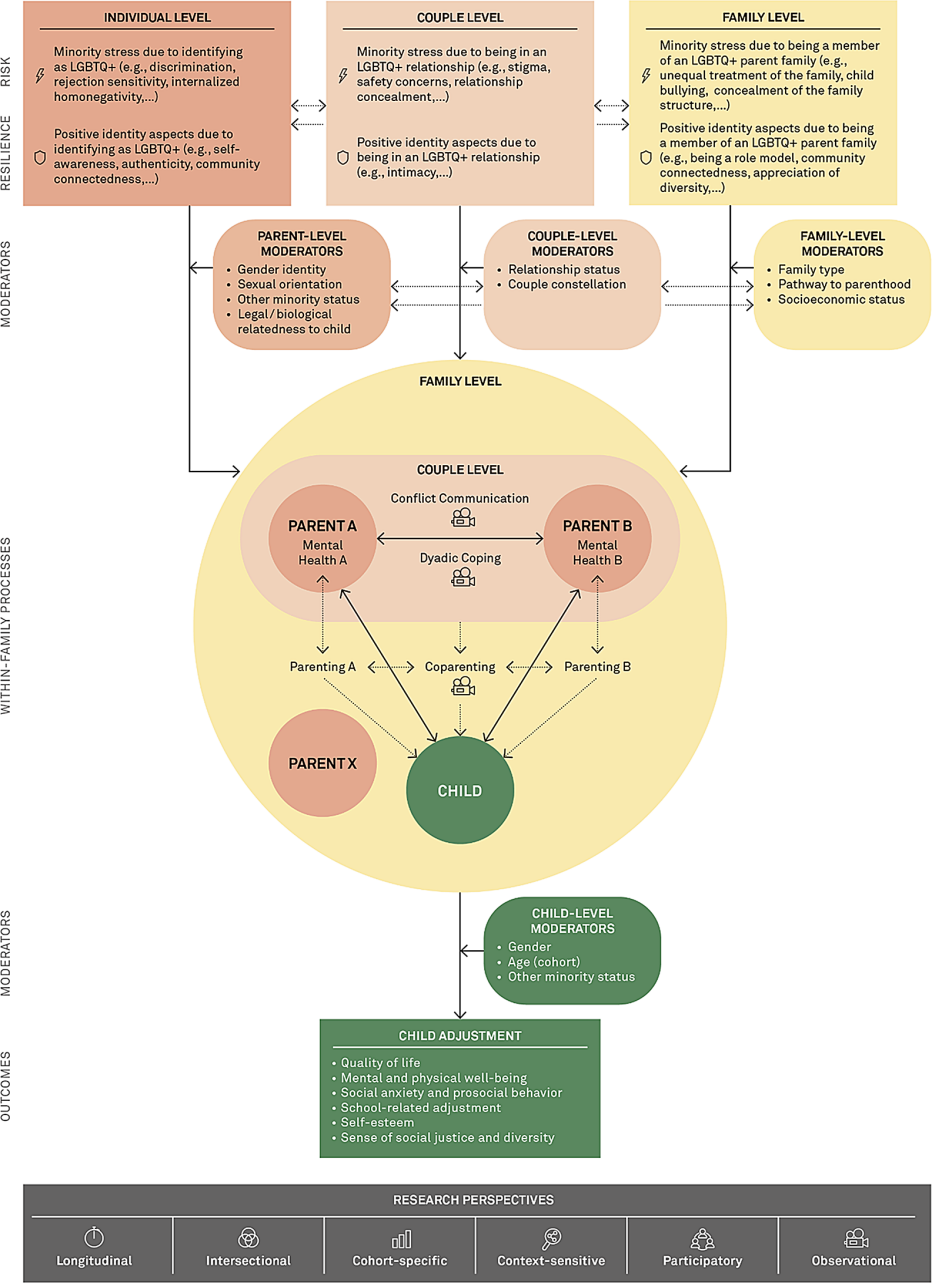
Study framework for risk and resilience in LGBTQ+ parent families

Figure 1 depicts our study framework based on theoretical contributions to LGBTQ+ parent family functioning [7, 19, 48], as well as prominent theories in LGBTQ+ health [28, 29, 33] and general family research [40, 53, 58, 115]. This framework (i) formalizes minority-specific predictors (both risk and resilience factors) and moderators of child and parent well-being in LGBTQ+ parent families by mapping them on multiple levels (i.e., the individual, couple, and family level), (ii) illuminates the role of within-family processes as potential key mechanisms, and (iii) includes overarching research perspectives (i.e., longitudinal, intersectional, cohort-specific, context-sensitive, participatory, and observational data).

The family unit is central to the model. It schematically consists of a child with two focal parents A and B (two people taking on parenting responsibilities, irrespective of legal or biological ties and regardless of whether a romantic relationship exists between them) [48] and any other parental caregivers (parent X). Siblings as well as single parents are considered in this model but not depicted for clarity. Following family systems theory [115], family members are mutually dependent subsystems, with their own as well as shared risk and resilience factors as a couple (parents only) and as a family (all members).

Positioned at the top of the model, minority-specific risk factors represent stressors that uniquely affect LGBTQ+ individuals, couples and families, ranging from discrimination (distal stressors) to internalized negative beliefs (proximal stressors). These stressors have been identified as having a significant impact on individual well-being and may show similar effects at the couple and family level (see section LGBTQ+ parent families: Parenting with pride and peril). This model further proposes that these factors may also impact child adjustment transdiagnostically, directly or indirectly, for example through the interparental relationship and coparenting. In contrast, minority-specific resilience factors, such as positive aspects related to an LGBTQ+ identity (e.g., self-awareness, authenticity), being in an LGBTQ+ relationship (e.g., intimacy), and the shared identity as an LGBTQ+ parent family (e.g., being a role model, community connectedness) [19, 41] may serve as protective factors. While their effects at the individual and couple level are well-established, future research is needed to explore how positive minority-specific identity aspects interact within the family system.

The center of the model is constituted by family processes, which include couple and parent-child interactions/relationships and coparenting, all of which contribute to family well-being. It has been suggested that these processes could be affected by minority-specific risk and resilience factors directly as well as indirectly through their influence on parental mental health [19].

At the bottom of the model, child outcomes are located, which are conceptualized as indicators of children’s psychological adjustment and well-being (i.a. quality of life, internalizing and externalizing problems, school related adjustment, social adjustment, self-esteem). We assume that many of the predictors and mechanisms postulated in the model act transdiagnostically [83], that is, they are relevant to a variety of child outcomes in the sense of multifinality [52].

Throughout the model, moderators of these associations, represented in the upper and lower sections, reflect the intersectional perspective guiding our work. These moderators are similarly located at multiple levels and moderate the effects of minority-specific risk and resilience factors on child adjustment, again through direct and indirect pathways [7, 48].

Beyond the immediate family environment, extra-family processes play a central role in our context-sensitive framework, shaping the environment in which family processes unfold. To account for these influences, we incorporate a measure for school climate to understand how educational institutions respond to and accommodate LGBTQ+ parent families. Moreover, extra-family processes inherently inform assessments on the individual, couple, and family level, such as stress related to unequal family recognition or safety concerns, and coping with everyday stressors.

### Sample and eligibility criteria

We aim to recruit *N* = 150 German-speaking LGBTQ+ parent families (i.e., participation of at least one LGBTQ+-identified parent) living in Austria, with at least one child aged 2;0 to 17;11 years. To be considered eligible, at least one parent in the family must identify as LGBTQ+ and children must have lived with at least one parent for at least two years (relevant for adopted or foster children). We have no restrictions regarding family type and consider parents to be anyone who takes responsibility for a child, regardless of genetic, gestational, or legal ties [48].

To simplify the analysis and differentiate between developmentally and societally significant age groups of children, families with children from three age cohorts will be recruited based on the child’s age at W1: (i) *early childhood*: child aged 2;0–5;11 years (target family sample size of *n* = 60); (ii) *middle childhood*: child aged 6;0–10;11 years (*n* = 50); (iii) *adolescence*: child aged 11;0–17;11 years (*n* = 40). These age groups were chosen to create developmentally homogenous groups of children and ensure the use of developmentally sensitive questionnaires and observational tasks (see Table 1). The sample sizes listed represent the target minimum sample sizes for each age cohort.

**Table 1:** Measures across age cohorts and data waves

Age Cohort	Wave 1 (W1)	Wave 2 (W2)	Wave 3 (W3)
Early childhood: Focal sample	*Child age: 2;0–5;11 years* Sociodemographic characteristics and moderator variables (parent-report): (a) *Parent individual level*: Gender identity, sexual orientation, age, race/ethnicity/migration history, other minority identity, biological and legal relatedness to child (b) *Couple and family level*: Relationship status, family-building history, family type, socioeconomic status (c) *Child level*: Age, gender identity, number of caregivers, race/ethnicity/migration history, other minority identity General questionnaires (parent-report): (a) *Parent individual level*: Adaptation of the Short Form Health Survey (SF-12) [116]; Patient Health Questionnaire (PHQ-2) [117]; Generalized Anxiety Disorder-2 (GAD-2) [118]; Lebenszufriedenheit-1 (L-1) [119] (b) *Couple level*: Couples Satisfaction Index (CSI-4) [120]; Dyadic Coping Inventory (DCI-SMS-K; based on SGM-CC) [121, 122] (c) *Family level*: Adaptation of the Parental Stress Questionnaire (ESF) [123]; Adaptation of the Coparenting Inventory for Parents and Adolescents (CI-PA) [124]; Brief Assessment of Family Functioning Scale (BAFFS) [125]; Family-friendly workplace (self-developed) Minority-specific questionnaires (parent-report): (a) *Parent individual level*: Adaptation of the Daily Heterosexist Experiences Questionnaire (DHEQ) [126]; Adaptation of the Lesbian, Gay, and Bisexual Identity Scale (LGBIS) [127, 128]; Lesbian, Gay, Bisexual Positive Identity Measure (LGB-PIM) [19, 42, 45, 46] (b) *Couple level*: Adaptation of the Couple Level Minority Stress Scale (CLMS) [38, 39]; Lesbian, Gay, and Bisexual Positive Identity Measure subscale *Intimacy* Measure (LGB-PIM) [45, 46]	*Child age: 3;0–6;11 years* Questionnaires same as W1 + new items from community-based participatory research (CBPR)	*Child age: 4;0–7;11 years* Questionnaires same as W2 + new items from CBPR
	(c) *Family level*: Daily Experiences with Heterosexism Questionnaire subscale *Parenting* (DEHQ) [126, 127]; Family Pride (self-developed based on [19]; Intrafamily Rejection Sensitivity (self-developed); Intraminority Family Stress (self-developed) (d) *Extra-family (i.e.*,* context) level*: School climate (self-developed for Austrian context; adapted from [129, [130] Questionnaires assessing child outcomes (parent-report): KIDSCREEN Quality of Life Measure in Children and Adolescents [131]; Pediatric Symptom Checklist (PSC-D) [132, [133] Observational assessment I (behavioral data): (a) Triadic interaction: Coparenting: Family Alliance Assessment Scales (FAAS) [134] (b) Dyadic interaction: Dyadic coping: Verhaltensbeobachtungssystem zur Erfassung des Dyadischen Copings (SEDC) [135] Additional questionnaires for subpopulations (parent-report): (a) Cisgender and heterosexual identified family members: Adaptation of the Lesbian, Gay, Bisexual Affiliate Stigma Measure subscale *Vicarious affiliate stigma* (LGB-ASM; [136]; Affiliate Pride (self-developed; adapted from [137]) (b) Single parents: Specificities of being an LGBTQ+ single parent (self-developed) (c) Separated/divorced parents: Reasons for separation, post-divorce arrangements (self-developed) Additional caregivers: Role in the family system and coparental arrangement		Observational assessment II see W1
Middle childhood	*Child age: 6;0–10;11 years* Sociodemographic characteristics and moderator variables (parent-report) Questionnaires see focal sample, Wave 1	*Child age: 7;0–11;11 years* Questionnaires same as W1 + new items from CBPR	*Child age: 8;0–12;11 years* Questionnaires same as W2 + new items from CBPR
**Age Cohort**	**Wave 1 (W1)**	**Wave 2 (W2)**	**Wave 3 (W3)**
Adolescence	*Child age: 11;0–17;11 years* Additional questionnaires for this age cohort (in addition to the focal sample): Sociodemographic characteristics and moderator variables (youth self-report) General questionnaires (youth self-report): (a) *Family level*: Adaptation of the Coparenting Inventory for Parents and Adolescents (CI-PA) [124]; Adaptation of the Elternbildfragebogen (EBF-KJ) [138]; Family Functioning: Brief Assessment of Family Functioning Scale (BAFFS) [125] Minority-specific questionnaires (youth self-report): (a) *Family level*: Adaptation of the Rainbow Family Scale (RFS) [139]; Family Pride (self-developed based on [19]) Questionnaires assessing child outcomes (youth self-report): KIDSCREEN Quality of Life Measure in Children and Adolescents [131, 132]; Pediatric Symptom Checklist (PSC-D) [132, 133]	*Child age: 12;0–18;11 years* Questionnaires same as W1 + new items from CBPR	*Child age: 13;0–19;11 years* Questionnaires see W2 + new items from CBPR

Families of the youngest age cohort (*early childhood*) represent the focal sample of our study and are invited to our lab at two time points (at W1 and W3), where observational data of key family process variables (e.g., dyadic coping and coparenting) will be collected using standardized interaction tasks (see section Procedures). This focal sample was chosen based on the expectation that the timing of family formation in combination with recent legal changes in Austria may have resulted in an increase in young LGBTQ+ parent families following the latest developments in adoption rights in 2016 (e.g., granting the right to joint adoption). Additionally, a focus on this age group allows for the examination of a crucial development period for the influence of family process variables to later health outcomes [52] and supports the use of standardized observational measures for a homogenous group. The lower age limit was set at 2;0 years, as we exclude the period of transition to parenthood and due to methodological reasons (no comparable outcome measures for infants available).

#### Sample size justification

Sample size determination in studies with hard-to-reach populations (such as LGBTQ+ parents) must necessarily strike a balance between the desired precision of effect estimation and the feasibility of recruiting the target sample size [140, 141]. This is compounded in our case, as a representative sampling frame for LGBTQ+ parent families in Austria does not exist and their exact number is unknown [108]. Our determined sample sizes of *n* = 150 index parents for the full sample (totaling 450 observations over three waves), and *n* = 60 for the focal sample (observational data; totaling 120 observations per family per wave; 240 observations total) are thus based on statistical, clinical, and recruitment-related considerations: Statistically, standards for precise sample size determination for complex, non-linear machine learning models have not been established yet [87]. We thus base our sample size determination on a series of available suggestions [141] that recommend a minimum number of 50 observations in total [142] and a minimum number of 10 observations per degree of freedom (i.e., model predictor) [143]. Our target sample sizes, number of observations, and all our main models meet these minimum requirements (50 to 140 observations). Traditional power analyses using the most conservative number of observations further reveal that the detection of small-to-medium effect sizes of *r* >.23 (*n* = 150) and *r* >.35 (*n* = 60) is possible with 80% power (two-tailed; *α* = 0.05). Similarly, 135 observations are required for detecting a medium-sized global effect of *f*^2^ = 0.15 [144] in a regression model with 14 predictors (cf. RQ 1; full sample). Thus, we will be able to detect bivariate and global effect sizes typically found in family psychology [145], sexual and gender minority health [146], and psychology at large [147] in our full sample, even when using more traditional modeling techniques. This also includes our most distal association with child outcomes (i.e., parental minority stress; estimated at *r* =.26 via ad-hoc meta-analysis based on [19]). Even in our focal sample (*n* = 60), clinically relevant effects can be detected with sufficient accuracy [145]. Regarding recruiting, our target sample size is well-supported by comparable or even larger recruitment efforts in Austria and other countries with similar population sizes (e.g., Israel, Finland), as well as our community collaborators. In all, our target sample sizes are justified in terms of reasonable power for the use of machine learning and more traditional statistical modeling techniques, while also being feasible in terms of recruitment.

### Procedures

#### Recruitment and remuneration

##### LGBTQ+ community services and associations

‘FAmOs’, a large association for LGBTQ+ parent families with local groups throughout Austria and our ongoing collaboration partner, will support us in publicizing the study, (e.g., via social media and in their Viennese Rainbow Family Centre), and recruiting among their members. Through our research (e.g., 42), we have also established a network with other LGBTQ+-related organizations that we will contact to serve as study multipliers. Printed study flyers will be disseminated at other LGBTQ+ and general family-related facilities in Vienna and surrounding federal provinces, as well as in listservs, newspapers, and magazines. Information about the study will additionally be shared broadly via social media posts (Facebook, Instagram, Twitter etc.), targeting LGBTQ+ individuals through digital flyers and project videos. Participating families are also asked to invite family friends or acquaintances who are eligible for inclusion, with 10 € as compensation per eligible new family (max. three times per family to avoid the clustering of individual friendship networks). Lastly, project staff and faculty colleagues will be asked to approach eligible families through their personal and vocational networks. Recruitment for the first wave will be completed by fall 2025.

##### Incentives

All participants will be compensated by a total of 50 € per family (for W1), 60 € (for W2), and 70 € (for W3). Participants of the focal sample will receive additional 50 € for the observational assessments in the lab at W1 and W3. Each child participating in the observational assessments will get an age-appropriate toy and each adolescent completing the questionnaires will receive a cinema voucher per wave.

CAB members are offered renumeration for their contribution as advisors. Each member will receive 50 € per person and meeting for the annual meetings at each data wave. Moreover, vouchers valued at 20 € will be offered to CAB members providing feedback on an initial version of the online questionnaire for the first data wave.

##### Sample retention and dropout prevention measures

Longitudinal studies with LGBTQ+ parents typically have high retention rates (e.g., 96–100% over five years) [60]. The still ongoing US-based National Lesbian Family Study [148] has shown how these high rates (92% after 35 years) can be attained by facilitating participation and providing a study identity. We incorporate this by providing easily accessible online surveys, ensuring consistent and accessible contact partners throughout the study, and framing our study using a name (i.e., RALF), accompanied by a logo, a website, and ongoing social media activity. Regular study newsletters, new year cards, and thank-you certificates will be sent to all participating families to ensure consistent contact. Besides monetary incentives, we expect that the participatory research components (see Table 2) will particularly increase participants’ commitment across data waves, as they can actively contribute to the project and follow study results easily and comprehensibly via the open access data explorer *ExploRALF*.

**Table 2:** *The RALF community-based participatory research framework*

Component	Description and implementation	Goals
Community advisory board (CAB)	CAB kickoff meeting (before W1): Focus group interview about the planned research project and study goals, systematically evaluated using thematic analysis [154]. Following recommendations [149], we will strive for transparency with CAB members regarding pre-planned, unchangeable procedures (e.g., observational tasks), while making adjustments based on CAB feedback where possible (e.g., questionnaires, recruitment procedure, study materials). A community advisory board (i.e., the RALF CAB) is formed, composed of volunteers from the LGBTQ+ community, study participants (parents and adolescents of LGBTQ+ parent families), community stakeholders (representatives of LGBTQ+ services and associations), and research staff members. Annual CAB meetings after completion of each data wave: Qualitative data (i.e., meeting notes) are reviewed by research staff to develop a summary of CAB activities that are implemented in future data waves (CBPR adaptations to the original study design). Short assessment tools (self-developed rating scales) are used that allow CAB members to evaluate different aspects of their work (e.g., how successful and effective they rate their efforts) and to provide their feedback on CAB integration continuously throughout the project. CAB members are offered fair pay for their contribution as advisors (i.e., 50 € per meeting for each person). Additionally, each CAB member who provides feedback on the W1 questionnaire will receive a 20 € voucher.	-) Establish a partnership based on mutual respect while addressing power differentials transparently [149] -) Collaborative participation by community, stakeholders, and academic partners -) Reciprocal capacity building and co-learning between researchers and community members to increase collective knowledge throughout the research project
Regular participant feedback	Open-ended questions about the following topics are presented at the end of each data wave in the online questionnaires: (1) feedback on study procedures, materials, and measures of the current wave; (2) suggested changes or improvements for future waves; (3) neglected/lacking research topics. Answers of the participants will be evaluated by the RALF CAB in the annual meetings and subsequently implemented where feasible.	-) Respectful and reciprocal interaction with study participants -) Integrate experiential knowledge of the study sample into scientific knowledge -) Use of measurements and wording sensitive to the participants
Community events	Two public events for the study participants, community stakeholders, CAB members, and the interested public: (1) Mid-project event (after W1): The event will be used to present preliminary results, discuss the interpretation and representativeness of the initial data, promote exchange between the participants, and share project experiences. (2) Closing event (after W3): We will share and discuss the main results of the project, host a panel discussion with the participating families and community stakeholders, and thank all participants for their participation. Attendees can provide their feedback about the events in the form of a qualitative evaluation.	-) Community involvement and engagement -) Sharing knowledge and experience in an accessible way -) Enhance relevance and acceptance of the research within the community
ExploRALF	Development of an interactive, intuitive, and open access web-app via *R Shiny* (research team; hosted on university server) to allow for low-threshold and easy-to-understand exploration of study results. Implementation of (1) filter sets (e.g., child age cohort; federal province) to allow for flexible disaggregation of data, and (2) exportable graphs and tabular results ready to use for community organizations. Feedback from the RALF CAB: Ensures usability, offers an opportunity for community members to be involved in dissemination efforts. Study participants are informed about how to use the ExploRALF.	-) Make research data accessible to the general public in an understandable way -) Achieve a community-friendly and transparent dissemination -) Ensure that the community is aware of and can benefit from study findings

#### Online questionnaires

We will provide parents and eligible adolescents with online self-report questionnaires, made available via SoSci Survey [134], with paper-and-pencil versions obtainable upon request. Data from each of the three yearly waves is matched using unique codes, which serve to pseudonymize the data and thereby guarantee the confidentiality of the participants.

#### Observational assessment

The observational data collection will focus on a triadic interaction (coparenting; between parents and a child) and a dyadic task (dyadic coping; between parents). In order to complete these two tasks, two caregivers must be determined for each participating family from the focal cohort. It is not a prerequisite, however, for the caregivers to be two legal, genetic or gestational parents in a romantic, dyadic relationship; rather, it is essential that both caregivers have a strong bond with each other and with the participating child. If two siblings in a family are of suitable age (two to five years), the younger sibling will be selected to participate. To complete the observational tasks, eligible families will be invited to our observational lab at W1 and W3. The lab is equipped with a video recording system, including state-of-the-art video (four dome cameras) and audio (stationary and portable microphones) recording devices. Software will include Media Recorder v6 (manufacturer: Noldus Information Technology BV) for synchronization of video and audio as well as Observer XT v15 (manufacturer: Noldus Information Technology BV) for coding, analysis, and processing of observational data.

##### The triadic interaction task – coparenting

We will use a standardized triadic interaction task, the *Lausanne Trilogue Play*, to assess coparenting. It consists of an approximately 10-minute-long free play session that is divided into four parts, each representing different relational constellations. In the first two parts, each caregiver plays with the child individually, while the other is present without interacting. Following that, both caregivers and the child play together in the third part. Finally, in the fourth part, only the caregivers converse while the child is not directly engaged. The examiner does not indicate to the caregivers when it is time to proceed to the next part of the task; rather, the caregivers themselves determine the exact duration of each of the four interactions.

The task is conducted within the laboratory environment, with the participants, two caregivers and a child, seated. The arrangement of caregivers’ and toddler’s seats forms an equilateral triangle (angle 60°) around a round table. The task will be audio- and video-recorded using the four stationary dome cameras within the laboratory setting, filming caregivers and children from every corner of the room. Within the laboratory setting, a clock is positioned in a visible location for the caregivers, serving as a reference, as they determine the duration of each of the four interactions.

##### The dyadic interaction task – dyadic coping

We will use a dyadic task to assess the adults’ dyadic coping with general and minority-specific stressors, as adapted from Bodenmann. Before starting their interaction, caregivers independently rate minority-specific (e.g., internalized stigma or barriers to starting or expanding a family) and general (e.g., work-related problems or financial difficulties) daily stressors on a 4-point-Likert scale (*a little stressful* to *very stressful*), facilitated by a member of the research team. Stressors must not include relationship problems, as this may shift the focus towards couple conflicts.

Following a rating of stressors, the most stressful issue that caregivers feel comfortable discussing is identified for each participant and topic pool (general vs. minority-specific). The first participant is randomly determined (coin toss) and asked to discuss the chosen topic for minority-specific stressors in a dyadic setting for eight minutes, while being videotaped. Subsequently, the second person discusses their chosen topic for eight minutes. A second coin toss determines the order of the following round of discussions about the most stressful general topic. Each participant will again be allotted eight minutes for this purpose. Although the second part of the task is intended to address general stressors, participants can discuss minority-specific stressors again, if they deem it more relevant. This approach acknowledges that minority-specific stressors may be an inherent part of the everyday lives of LGBTQ+ individuals and thus may at times be indistinguishable from the general stressors participants encounter. As such, participants have the liberty to address minority-specific stressors in greater depth, if they wish, or focus exclusively on general stressors in the second part instead. Conversely, participants may choose to focus solely on general stressors in both parts of the task, should they feel more comfortable doing so.

During the discussions, neither the child nor the examiner will be present in the room. The task takes place in a laboratory setting, with both participants seated close enough to interact physically and maintain eye contact. There will be no objects for participants to hold during the task, and no table in between them as a barrier. The session will be audio- and video-recorded using the four stationary dome cameras within the laboratory setting, filming partners from every corner of the room.

### Measures

Table 1 provides an overview of the measures across age cohorts and data waves in which they are applied.

#### Questionnaire data

Before completing the questionnaire, participants will answer preliminary questions used to tailor the wording of items and scales to their preferred terminology. For instance, each questionnaire will be adapted in accordance with specific identity characteristics, such as pronouns or partner terminology. This is achieved by using placeholders in the online survey, which allows for participants’ inputs to be inserted into the questionnaire.

All adult participants will answer general questions, while minority-specific scales are either universal to the population of LGBTQ+ parent families (e.g., LGBTQ+ parent family pride) or tailored to specific sub-populations (e.g., the specificities of being an LGBTQ+ single parent) and will only be completed by participants to whom they are relevant. Additionally, parents report on child outcomes, such as well-being and academic adjustment. To provide a supplementary perspective on adolescent well-being, self-report questionnaires will be completed by adolescents aged 11 years or older. These age-sensitive questionnaires assess their well-being as well as adolescents’ perception of their parents’ coparenting along with minority-specific experiences related to being part of LGBTQ+ parent families. The adolescent version of the questionnaire underwent thorough scrutiny to ensure its age-sensitivity and concision.

Wherever feasible, we utilized established scales that already have published German language versions. In the absence of a German translation for certain scales, two members of the team translated and adapted the questionnaires to the Austrian research context and reconciled differences with the translations until consensus throughout the project team was reached.

#### Coding of observational data

##### The triadic interaction task – coparenting

The *Family Alliance Assessment Scale* (FAAS) will be used to code triadic interactions based on nonverbal interaction cues to rate family engagement and coordination of joint activities across 15 scales. The scales measure seven principal interactive functions: affect sharing, child subsystem, coparenting, focalization, participation, organization, timing/synchronization. Each scale uses a 3-point ordinal scoring system (2 points = *appropriate*, 1 point = *moderate*, 0 points = *inappropriate*), with total scores ranging from 0 to 30. The FAAS has been shown to have acceptable reliability and validity. Coding of full videos will be conducted by two independent raters, trained extensively within the FAAS coding system to reach acceptable reliability prior to coding of study videos. Inter-rater reliability will be assessed using intraclass correlation coefficients. Coding disagreements will be resolved by consensus.

##### The dyadic interaction task – Dyadic coping

Data resulting from the dyadic interaction task will be analyzed using the *System to Evaluate Dyadic Coping* (SEDC) [135]. Data is coded according to the type of coping behavior displayed by the dyad. This includes verbal and nonverbal supportive dyadic coping (e.g., validating partner, listening attentively and showing interest, as well as nonverbal, problem-focused and emotion-focused dyadic coping) and negative dyadic coping (e.g., ignoring a partner’s stress communication, ambivalent, hostile or superficial dyadic coping). The interactions in each of the four 8-minute parts of the task are rated at 10-second intervals for support interactions, totaling 48 sequences, for occurrences of dyadic coping categories (e.g., 0 = *did not occur*; 1 = *did occur*). This coding system has been validated [150] and employed in the context of LGBTQ+ research, with findings indicating adequate reliability [151]. Coding will be conducted using two independent raters trained extensively within the SEDC prior to coding study videos. Following typical procedures employed for SEDC coding (e.g., [152]), coders will code both partners in 10% of videos to assess interrater reliability. Within the remaining videos, coders will code only one of the partners (random assignment).

### Community-based participatory research components

A CAB is established to accompany and advise the study throughout its entire duration. To represent the experiences of different LGBTQ+ parent families and the perspectives of the community, the RALF CAB is made up of nine community representatives, appointed in cooperation with ‘FAmOs’. The advisory board includes ‘FAmOs’ members as well as parents and children aged 14 and over from LGBTQ+ parent families. Members will attend the CAB meetings at three timepoints, occurring at one-year intervals, throughout the course of the study. Meetings are scheduled to precede the three data waves, with the objective of integrating the information gained from these meetings into the respective data collection. During CAB meetings, semi-structured interviews are utilized to address topics that are important for the course of the study and the participants’ experiences. For instance, discussions may focus on the phrasing of items or their applicability, the research design or execution of observational tasks.

Audio data collected at each CAB meeting will be transcribed by an automatic transcription service suitable for German language transcription, *MAXQDA Transcription* [153], available within the software. Transcription will be reviewed by the team until consensus is reached and analyzed using thematic analysis [154] further supported by MAXQDA [153]. In analyzing the data, we will focus on recommendations made by the CAB members for the study. To ensure the effective implementation of the results, we introduce a review process, requiring CAB members to complete a short assessment after each meeting. These assessments determine the perceived success and effectiveness of members’ efforts as well as the relevance and the feasibility of the changes discussed during the meeting.

The RALF CAB, including its member feedback, plays a central role in ensuring that the voices and perspectives of the community are meaningfully integrated into the study. Beyond that, community engagement is further strengthened through additional CBPR elements, such as participant feedback, community events, and the *ExploRALF*, a data explorer of study results. Specifically, upon completion of the online questionnaire and observational study of each data wave, participants will be asked to suggest changes, indicate topics that may not have been sufficiently addressed yet, and provide feedback on the methodology and user-friendliness of the study through open-ended questions. The responses will be evaluated at the following CAB meeting and implemented into the subsequent data wave, when applicable. Two public events, scheduled at the midpoint and upon completion of the third wave, will serve as platforms for dialogue between researchers, participants, community stakeholders, and the wider public, providing further opportunities to discuss findings and foster collaborative exchange.

In addition, the *ExploRALF*, an interactive open access web-application, will make study results widely accessible. Designed as a user-friendly and transparent tool to showcase results, it will allow anyone to interactively explore anonymized data, apply custom filters and visualize findings on the RALF project website. To maximize its impact and usability, participants will receive guidance on how to navigate the *ExploRALF* effectively, allowing them to fully engage with and benefit from the study’s findings. The incorporation of CBPR elements in the RALF study supports the meaningful integration of diverse, intersectional perspectives in the study, thereby enhancing the relevance and validity of the resulting data. Table 2 provides a comprehensive overview of the CBPR elements used in the RALF study.

### Data analysis

We will utilize GBDT models using the LightGBM framework [84] for all ML analyses. GBDT models were selected for their ability to handle multicollinearity, incorporate high-dimensional variable interactions, and capture non-linear associations between predictor and outcome variables [84], aligning with the intersectionality-informed theoretical framework of our analysis.

To prevent data leakage, mitigate overfitting and improve generalizability, we will apply cross-validation [155, 156]. Specifically, we will implement *nested* cross-validation, which has been shown to be particularly valuable in the context of small samples. By producing robust performance estimates independent of sample size, this approach addresses the common limitations of predictive accuracy in such settings [155]. To avoid potential overfitting to family clusters resulting from the dependency of data among individuals within the same family, we include family ID as a grouping factor in all analyses. Within the inner cross-validation loop, we will conduct hyperparameter tuning to optimize model performance. We will evaluate model performance using prediction *R*^*2*^ and mean absolute error for regression settings and balanced accuracy for classification settings [156]. SHAP (Shapley additive exPlanations) values will be used to assess individual predictor contribution [157] in combination with partial dependence plots [158]. To evaluate the statistical significance of the prediction *R*² and the independent variables’ importances, we apply a modified *t*-test that accounts for the dependency introduced by cross-validation [159, 160].

We will use the following dependent and independent variables as model input: For RQ 1, child adjustment (e.g., PSC full score) will be predicted from child age and gender; parental sexual orientation, gender identity, mental health, minority stress, positive identity aspects; parenting, pathway to family formation, number of caregivers, family type, and socioeconomic status. This analysis will be conducted cross-sectionally at each wave, as well as after completion of all three waves, taking participant nesting within waves into account.

For RQ 2, we will first predict child adjustment (W3) by parental minority stress (W1), parental mental health (W2), and time-related (minority stress at W2, W3; parental mental health at W1, W3; child adjustment at W1, W2) as well as sample-related covariates (child age and gender at W3; parental sexual orientation and gender identity at W1). Second, we will probe longitudinal associations between minority stress and parental mental health by fitting a simpler model predicting parental mental health (W2, W3) by parental minority stress at the previous wave and relevant covariates.

For RQ 3, we will first fit separate models for dyadic (dyadic coping) and triadic (coparenting) family processes at W3 as outcomes, and minority stress (W2), minority stress (W3), parental gender identity, and parental sexual orientation as covariates. A separate series of models will be fit for positive identity aspects instead of minority stress as the predictor of interest. We will then probe longitudinal associations between family processes and child adjustment by predicting child adjustment at W2 by family processes (coparenting, dyadic coping), child gender, child adjustment, and parental couple gender at W1. We will use data from parents identifying as LGBTQ+ for the first series of models (*n* ≥ 60) and data from all parents (including heterosexual) for the second series of models (*n* = 120).

### Dissemination

Study results will be disseminated in national and international scientific journals and conferences. To ensure accessibility beyond academia, the project’s main results will be published and distributed to the public in a comprehensible way (e.g., press announcements, social media). Additionally, we will host two public events, bringing together study participants, community stakeholders, and the interested wider public. These events serve to present and discuss (preliminary) results, promote exchange between the participants, and provide an opportunity for participants to share their experiences.

To further support transparent and interactive engagement with our findings, we will develop the *ExploRALF*, which makes anonymous study results publicly available. The *ExploRALF* will allow for disaggregated data analysis of selected items by using a set of individualized filters. This approach has been proven of great applied value to community organizations in previous collaborations [161] due to their need for flexible, easy-to-use analysis tools and is deployable through the open-source software *R Shiny* and accompanying hosting solutions.

### Adherence to open science principles

Committed to the FAIR (findable, accessible, interoperable, and reusable) principles of open science, we will make anonymized data, analysis scripts, outputs as well as preprints and open access versions of final publications available to the research community via OSF. In addition, individual publications resulting from the RALF study data will be preregistered separately.

Beyond being archived, the fully anonymized data will serve as the basis for the *ExploRALF*. Study results can be viewed through univariate statistics (e.g., frequencies, central tendency, and dispersion measures) or visualized through interactive charts, whereby results can be displayed adaptively for subgroups (e.g., bisexual parents) using various filter options. With the integration of these open science practices, we aim to increase transparency and broaden the utility of the results of the RALF study.

### Transparency statement regarding generative artificial intelligence (AI)

During the preparation of this manuscript, ChatGPT (versions 3.5, 4o) [162] and DeepL Write (free version) [163] were used on author-generated text [BG] to edit for grammar, clarity, and brevity, only. Editing suggestions were thoroughly reviewed and implemented if they were deemed suitable. However, no original texts, ideas or references were generated with the help of a large language model or any other generative AI. The authors of this paper take full responsibility for the content of this manuscript.

## Discussion

This study protocol introduces the RALF study, a national three-wave longitudinal study with three child cohorts of LGBTQ+ parent families in Austria. Combining questionnaire data with observational tasks, RALF aims to provide comprehensive insights into the well-being and family functioning of LGBTQ+ parent families with a focus on general and minority-specific risk and resilience factors across multiple levels of the family system, including the individual, couple, and family level. Research on LGBTQ+ parent families has historically been shaped by between-group designs comparing LGBTQ+ parent families to heterosexual cisgender families [6, 7]. Given that this generation of research has made a significant contribution to societal and legal acceptance [13, 15, 16, 20], scholars may now turn their attention on within-group designs to explore the complexity of risk and resilience factors that shape LGBTQ+ parent family life [1, 6, 7, 15]. This shift from comparative to within-group designs [15] has given rise to several new challenges in the field, particularly in quantitatively capturing the diversity of experiences within LGBTQ+ parent families, such as those related to experiences of marginalization and privilege, as well as to different family forms [18].

Accurately representing these experiences requires the development of comprehensive, theory-driven study frameworks and modeling techniques accounting for intersectional identities [15, 75, 80, 81], different relational levels within families [19], and broader extra-family context factors, such as societal and legal contexts that shape family outcomes [18, 48]. Innovative analytical methodologies, such as ML models, present a promising approach as they can capture the complex non-linear associations and high-dimensional interactions of these factors both concurrently and over time [81–84]. Within the RALF study, these models will be used to provide deeper insights into children’s developmental trajectories in LGBTQ+ parent families, the influence of intra- and extra-family factors on individual and family well-being over time, and the mechanisms that help buffer against or amplify risks in these contexts.

In Austria, where RALF is being conducted, another significant challenge lies in a lack of data on LGBTQ+ parent families [108]. Although recent legal and social developments [14, 110–112] point to a possible increase in LGBTQ+ parent families [113, 114], we still know very little about the well-being of the parents and children living in this country. CBPR offers a promising approach to bridge this gap by actively involving LGBTQ+ parent families in the research process. This actively collaborative approach helps ensure that the experiences and needs of communities, such as LGBTQ+ parent families, are reflected in study designs and findings, enhancing both the relevance and the applicability of results [97].

Building on these insights, the RALF study aims to address these challenges by incorporating a national, three-wave longitudinal design, a multi-method framework (self-report and observational data), and a multi-rater (parents and children) approach. This study framework allows for exploring both within- and extra-family processes, incorporating minority-specific factors as well as risk and resilience factors at multiple levels of the family system, namely the individual, couple, and family level. The inclusion of three child cohorts enables development-sensitive measures and age-specific analyses, while the longitudinal design offers insights into the interplay between family dynamics, minority-specific factors, and broader contextual influences over time. Advanced analytical, machine learning-based techniques, such as GBDT [84], in combination with intuitive measures for variable importance [157], allow for an intersectionality-informed modeling strategy involving complex data structures, while nested cross-validation addresses potential overfitting in small datasets, ensuring robustness and valid inferences [155]. Lastly, the RALF study is based on a comprehensive CBPR approach to ensure the findings align with the needs of LGBTQ+ parent families in Austria. The integration of CBPR into the research process is invaluable for fostering a meaningful mutual exchange with the community, thereby building trust and ensuring that the resulting research accurately reflects the community’s lived experiences [97, 100, 102, 103]. Tools such as the data explorer *ExploRALF* and community events will strengthen the study’s community relevance and facilitate dissemination of findings.

### Limitations

While RALF demonstrates several notable strengths, some already foreseeable limitations should be noted. First, the use of non-probability sampling (i.e., convenience and snowball sampling) for recruitment may limit the generalizability of findings [164, 165]. Thus, our sample will not be representative of the Austrian LGBTQ+ parent family population. To mitigate sampling bias and diversify our sample composition, we have implemented a multi-tiered recruitment strategy including outreach through cooperation with community associations, the RALF CAB, listservs, newspapers and magazines, as well as social media platforms and community events. Non-probabilistic sampling techniques are common in LGBTQ+ family research [20, 148, 166], including prominent studies like the EU LGBTI surveys conducted by the European Union Agency for Fundamental Rights (FRA) (e.g., [167]).

Second, some participant groups (e.g., trans and non-binary parents) might not be well-represented within our study due to a low base rate within the population, despite strong recruitment efforts to increase sample diversity. Thus, we might need to aggregate some subgroups (e.g., non-cisgender parents) to achieve sufficient power for stable modeling. This aggregation could, however, mask meaningful in-group diversity of the sample. Furthermore, the grouping process itself may introduce subjectivity, for example by miscategorization [168] or oversimplification.

Third, time-intensive assessments per time point and the longitudinal design harbor the risk of high dropout rates within and across data waves. We therefore hope to optimize our retention rate by providing incentives, such as monetary compensation, a strong social media presence, community events, and showcasing results in the accessible data explorer *ExploRALF*. Encouragingly, similar longitudinal studies involving LGBTQ+ parents have demonstrated high retention rates, ranging from 96 to 100% over five years [60].

## Conclusion

Taken together, the RALF study is the first comprehensive, longitudinal study to investigate the associations between minority-specific risk and resilience factors and the well-being and family functioning of LGBTQ+ parent families in Austria. The study addresses empirical gaps in the literature, such as underexplored within-family processes [7, 19] as well as contextual factors [9, 48, 169]. RALF also responds to the need to theoretically acknowledge and statistically model the diversity [1, 6, 9, 18, 64, 72, 169] and intersectional experiences [6, 18, 72, 170] within LGBTQ+ parent families. By making its findings accessible through low-threshold tools, such as the *ExploRALF*, and disseminating findings through public engagement events, the study aims to strengthen connections within the community and ensures that its results have a meaningful impact. Their practical implications have the potential to inform Austrian policy and clinical practice that support LGBTQ+ families in education, healthcare, social services, and beyond.

## Data Availability

No datasets were generated or analysed during the current study.

## Abbreviations

CAB: Community advisory board
CBPR: Community-based participatory research
FAAS: The Family Alliance Assessment Scale
GBDT: Gradient boosted decision trees
H: Hypothesis
LGBTQ+: Lesbian, gay, bisexual, trans, queer and other non-heterosexual sexual and non-binary gender identities
LG: Lesbian and gay
ML: Machine learning
MST: The minority stress theory
OSF: Open Science Framework
RALF study: The Rainbow Austrian Longitudinal Family study
RQ: Research question
SEDC: System to Evaluate Dyadic Coping
SHAP: Shapley additive values
W (1–3): (Data) wave
